# Lymphatic Invasion Acts as a ‘Hidden Risk Factor’: Four-Fold Increased Mortality Risk in Early-Stage (TNM Stage I, N0) Non-Small Cell Lung Cancer

**DOI:** 10.3390/jcm15124582

**Published:** 2026-06-12

**Authors:** Kadir Burak Özer, Suat Erus, Ezgi Cesur, Özgür Güzey, Pınar Bulutay, Serhan Tanju, Pınar Fırat, Şükrü Dilege

**Affiliations:** 1 Department of Thoracic Surgery, Koç University School of Medicine, 34010 Istanbul, Türkiye; kdrbrkozer@gmail.com (K.B.Ö.);; 2 Department of Thoracic Surgery, VKF American Hospital, 34365 Istanbul, Türkiye; 3 Department of Pathology, Koç University School of Medicine, 34010 Istanbul, Türkiye; pbulutay@ku.edu.tr (P.B.);

**Keywords:** non-small cell lung cancer, lymphatic invasion, overall survival, prognostic factor, adjuvant therapy, TNM staging

## Abstract

**Background/Objectives**: Despite advances in the TNM staging system, prognostic heterogeneity persists in early-stage non-small cell lung cancer (NSCLC). Lymphatic invasion (LI) is a known marker of aggression, but its independent significance in the critical, low-risk Stage I, N0 subgroup—typically ineligible for adjuvant therapy—remains poorly defined. We hypothesized that LI acts as a powerful, yet hidden, risk factor in this highly favourable cohort. **Methods**: This retrospective cohort study included 988 consecutive patients who underwent curative anatomical resection for NSCLC. All patients underwent complete resection with pathologically confirmed negative surgical margins (R0 resection). Cases were staged according to the 9th Edition of the TNM Classification of Malignant Tumours (TNM-9) and grouped as LI-positive or LI-negative. A critical subgroup analysis focused on 347 truly low-risk patients (TNM Stage I, N0, no vascular or pleural invasion). Overall survival (OS) was evaluated using the Kaplan–Meier method and multivariable Cox proportional hazards models. **Results**: In the entire cohort (*n* = 988), LI was present in 40.9% of cases. LI positivity was an independent predictor of worse OS in multivariable analysis (HR: 1.520, 95% CI: 1.004–2.301, *p* = 0.048). In the low-risk subgroup (n = 347), the presence of LI resulted in a drastic survival divergence, with 5-year OS declining from 96.1% (LI-negative) to 83.8% (LI-positive). Multivariable analysis confirmed LI as an independent adverse prognostic factor in this subgroup (HR: 4.002, 95% CI: 1.567–10.221, *p* = 0.004). **Conclusions**: Lymphatic invasion is a robust, independent adverse prognostic factor in resected NSCLC. LI may identify a subset of early-stage N0 NSCLC patients who warrant closer postoperative surveillance and prospective evaluation for adjuvant treatment strategies. Validation in prospective cohorts is required before LI can be formally integrated into staging algorithms or treatment guidelines.

## 1. Introduction

Globally, lung cancer continues to be a primary driver of cancer-related death. The high incidence and fatal outcomes associated with this disease necessitate continuous and in-depth research in oncology [1]. Cancer staging is a fundamental approach, applied since the early periods of medical history, aimed at defining disease extent and predicting its biological course. Staging allows for the prediction of anatomical disease spread and potential treatment response, thereby estimating patient survival and quality of life.

The most widely used system for non-small cell lung cancer (NSCLC) is the TNM staging system, which systematically evaluates the primary tumour size and invasion degree (T), regional lymph node involvement (N), and distant metastasis (M). While the TNM system is highly valuable, it has been updated numerous times due to changing diagnostic and treatment protocols over the years [2]. Nonetheless, it can often be insufficient in fully explaining prognosis, as significant differences in outcomes are frequently observed among patients within the same stage [3]. Studies focusing on specific early-stage subsets, such as p-T1aN0M0 adenocarcinoma [4] and Stage IB NSCLC [5], have repeatedly highlighted this prognostic heterogeneity, underscoring the necessity for better stratification tools.

Research has increasingly shown that differences in tumour aggressiveness and behaviour play a significant role in determining prognosis. Pathological features reflecting tumour aggression, such as visceral pleural invasion, have been incorporated into the TNM classification and play an essential role in adjuvant therapy decisions. Similarly, lymphatic invasion (LI) is recognised as another important prognostic factor [6]. LI has been established as an adverse prognostic factor for survival and a valid parameter for treatment selection in numerous malignancies, including breast, colon, and gastric cancers [7,8]. It is hypothesised that LI reflects tumour aggressiveness, acting as a critical first step in the metastatic cascade [9]. Furthermore, in gynaecological and head and neck tumours, adjuvant therapy is suggested even in cases of node-negative disease when LI is present [10,11].

However, the definitive role of LI in the prognosis and treatment planning of NSCLC remains controversial. The current body of knowledge is limited, leading to uncertainties regarding its impact on overall patient survival. Determining the independent prognostic significance of LI, particularly in early-stage (Stage I) N0 patients who, according to current guidelines, do not require adjuvant therapy, is paramount for the personalisation of clinical decisions.

Therefore, this study focuses on the potential effects of lymphatic invasion on the prognosis of NSCLC patients. We aimed to definitively establish the independent prognostic role of LI in NSCLC, specifically within the early-stage subgroup ineligible for standard adjuvant therapy, with the hope of providing a clearer understanding of its impact and potentially contributing to individualised treatment protocols.

## 2. Materials and Methods

### 2.1. Patient Selection and Study Design

This retrospective cohort study included a total of 988 consecutive patients who underwent anatomical resection (lobectomy or segmentectomy) for primary non-small cell lung cancer between February 2006 and September 2023. Archival surgical specimens were re-evaluated, and all cases were retrospectively divided into two distinct groups: lymphatic invasion-positive and lymphatic invasion-negative.

Exclusion criteria were patients who received neoadjuvant therapy for primary lung cancer and those who underwent non-anatomical resection (e.g., wedge resection). All included patients underwent complete resection with pathologically confirmed negative surgical margins (R0 resection). Patients with microscopically or macroscopically incomplete resection (R1 or R2) were excluded from the analysis. Any death observed within the first 90 postoperative days was classified as operative mortality.

### 2.2. Prognostic Subgroup Definition

The main focus of the study—the prognostic subgroup without indication for adjuvant therapy—was defined based on the 9th Edition of the TNM Classification of Malignant Tumours (TNM-9) and international guidelines. The following specific criteria were used to select patients for this critical subgroup analysis: tumour size less than 3 cm; absence of vascular and pleural invasion; pathologically N0 (lymph node negative) status; Stage I primary lung cancer; and absence of any documented adjuvant therapy. These two groups (LI-positive and LI-negative) were then comparatively analysed for tumour type, dominant histological subtype, nodal involvement status, tumour stage, and overall survival.

### 2.3. Histological Evaluation

**Figure 1:**
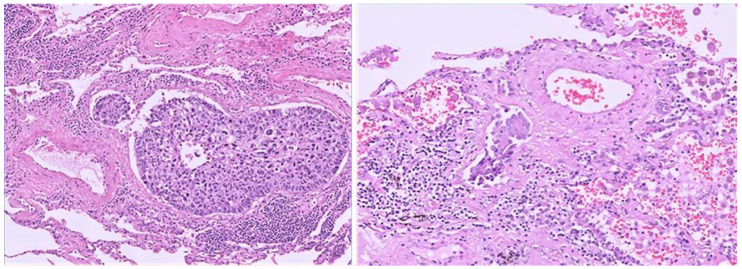
Representative histological image demonstrating lymphatic invasion (H&E staining). Tumour foci are visible within the lumen of lymphatic vessels located adjacent to the tumour.

The tumour-containing lung resection material was sliced and fixed in 10% formalin solution overnight. Following fixation, all tumours with a diameter less than 3 cm were entirely sampled, and for larger tumours, the maximum possible number of samples were taken, in accordance with guideline recommendations. The prepared sections were stained with haematoxylin-eosin (H&E) and independently assessed for lymphatic invasion by two experienced pulmonary pathologists (P.B. and P.F.). Lymphatic invasion was assessed exclusively using standard H&E staining in all cases; immunohistochemical markers such as D2-40 or podoplanin were not employed at any stage of the evaluation. Lymphatic invasion was defined as the presence of tumour foci, free or attached to the vessel wall, within the lumen of lymphatic vessels located inside or adjacent to the tumour. A case was considered “lymphatic invasion positive” if tumour foci were detected in at least one lymphatic vessel (Figure 1).

### 2.4. Statistical Analysis

Descriptive statistics were expressed as mean values ± SD or as number and percentage. Baseline characteristics between LI-positive and LI-negative groups were compared using the Chi-square test and Student’s *t*-test (or analysis of variance for continuous data). Survival data were analysed using the Kaplan–Meier method to estimate time-specific survival rates (2- and 5-year OS). The difference between survival curves was tested using the log-rank test. The independent prognostic significance of LI was evaluated using multivariable Cox proportional hazards regression models. For the entire cohort, the model included age, sex, histological type, tumour diameter, grouped pathological stage (Stage I/Stage II/Stage III–IV), extent of resection (standard vs. extended), and calendar period (2006–2014 vs. 2015–2023) as covariates. For the low-risk subgroup, the model included age, sex, histological type, and tumour diameter; calendar period was not included due to the limited number of events (*n* = 19), which precluded the safe addition of further covariates without risking model overfitting. A probability of *p* < 0.05 was considered statistically significant. All computations were performed using SPSS Statistics for Windows, version 26.0 (IBM Corp., Armonk, NY, USA).

### 2.5. Ethical Approval

The study protocol was approved by the Institutional Ethics Committee of Koç University (Approval Date: 20 November 2025; Reference Number: 2025.529.IRB2.251). The research was performed in accordance with the ethical standards laid down in the 1964 Declaration of Helsinki and its later amendments. Due to the retrospective nature of the study, the requirement for individual informed consent was formally waived by the Institutional Review Board. Patient data were fully anonymised before analysis.

## 3. Results

### 3.1. Patient and Tumour Characteristics

A total of 988 patients who underwent anatomical lung resection for NSCLC were included in the study. The study cohort demonstrated low perioperative risk, with 8 patients (0.81%) experiencing operative mortality. Lymphatic invasion (LI) was detected in 404 patients (40.9%). As shown in Table 1, the presence of LI was significantly associated with more aggressive tumour characteristics. Patients with LI-positive tumours demonstrated significantly larger mean tumour size (33.9 mm ± 20.0 vs. 26.7 mm ± 21.5; *p* < 0.001).

It should be noted that the single patient with pathological N3 disease in this cohort represents a case of false-negative mediastinal staging occurring in the pre-EBUS era of our series. Preoperative PET-CT demonstrated no suspicious lymph node uptake, and subsequent mediastinoscopy with intraoperative frozen section analysis of the contralateral station 4R lymph node returned a negative result, prompting proceeding with curative-intent pulmonary resection. N3 nodal involvement at station 4R was identified only on final pathological examination of the mediastinoscopy specimen, representing an unexpected pathological upstaging rather than intentional contralateral nodal dissection. The 18 patients with pathological Stage IV disease represent a carefully selected group of oligometastatic cases who underwent surgery as part of a curative-intent multimodality strategy without requiring neoadjuvant systemic therapy prior to pulmonary resection. The majority had undergone prior surgical resection of solitary intracranial metastases, with a minority presenting with solitary subcutaneous metastasis.

Furthermore, LI was a strong indicator of regional metastatic burden; while the majority of LI-negative patients presented with N0 status (87.8%), LI-positive tumours exhibited significantly higher rates of regional nodal involvement (N1, N2a, N2b, and N3) (*p* < 0.001). Specifically, the rate of N1 involvement was significantly higher in LI-positive patients (27.7%; 112/404) compared to LI-negative patients (10.3%; 60/584). More critically, the rate of N2a involvement in LI-positive patients (13.9%; 56/404) was substantially higher than that observed in LI-negative patients (1.7%; 10/584). LI was also significantly associated with a higher tumour stage at diagnosis (*p* < 0.001) (Table 1).

The relationship between LI and histological tumour type was analysed (Table 2). LI was observed in 38.1% (238/625) of adenocarcinoma cases and 43.1% (113/262) of squamous cell carcinoma cases (*p* = 0.017). When focusing on the adenocarcinoma subgroup (*n* = 625) and its distinct histological patterns (Table 3), a statistically significant relationship was found (*p* < 0.001). Among the classified cases, LI was observed most frequently in the solid (53.2%; 66/124) and acinar predominant patterns (42.9%; 105/245). Conversely, the lepidic pattern showed the lowest LI rate (19.4%; 12/62).

### 3.2. Overall Survival Analysis

**Figure 2:**
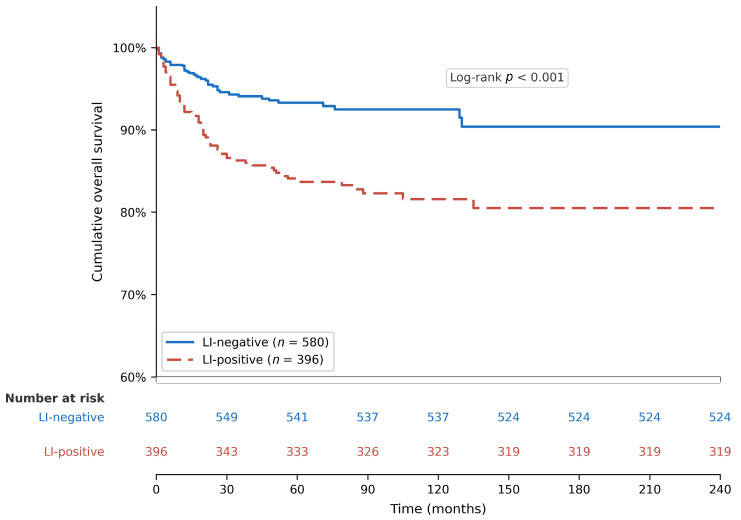
Overall survival curves based on the presence of lymphatic invasion in the entire cohort (*n* = 988), with numbers at risk. LI-positive patients demonstrated significantly reduced overall survival compared to LI-negative patients (log-rank *p* < 0.001).

The entire cohort was followed for a median period of 67 months (range 0–233 months). Of the 976 evaluable patients, 108 deaths were recorded during follow-up: 41 in the LI-negative group (*n* = 580) and 67 in the LI-positive group (*n* = 396). Overall survival (OS) outcomes were assessed utilising the Kaplan–Meier method. The Kaplan–Meier survival curves revealed that LI positivity was significantly associated with reduced overall survival across the entire cohort (log-rank test, *p* < 0.001; Figure 2). The 2-year OS rate was 95.3% for LI-negative patients compared to 93.3% for LI-positive patients, and the 5-year OS rate was 88.1% and 84.1%, respectively.

Multivariable Cox proportional hazards regression analysis, adjusting for age, sex, histological type, tumour diameter, pathological stage, extent of resection, and calendar period (2006–2014 vs. 2015–2023), confirmed the independent prognostic impact of LI on OS (HR: 1.520, 95% CI: 1.004–2.301, *p* = 0.048). Notably, calendar period was itself a highly significant prognostic factor (HR: 0.217, 95% CI: 0.145–0.326, *p* < 0.001), reflecting substantially improved outcomes in the more recent era. The complete multivariable model results are presented in Table 4.

### 3.3. Prognostic Role in the Early-Stage Subgroup

A predefined critical subgroup of 347 early-stage patients (TNM Stage I, N0, no vascular or pleural invasion, no adjuvant therapy) was isolated for specific analysis. Within this cohort, LI was detected in 55 patients (15.9%). Of the 343 evaluable patients, 19 total deaths were recorded during follow-up: 11 in the LI-negative group (*n* = 289) and 8 in the LI-positive group (*n* = 54).

**Figure 3:**
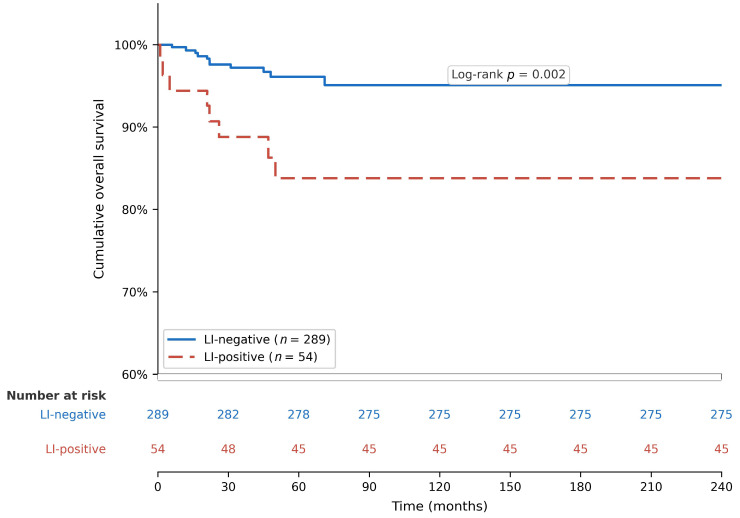
Overall survival curves based on the presence of lymphatic invasion in the low-risk subgroup (TNM Stage I, N0, *n* = 347), with numbers at risk. LI-positive patients demonstrated significantly reduced overall survival compared to LI-negative patients (log-rank *p* = 0.002).

LI remained a highly significant prognostic factor for reduced overall survival (log-rank test, *p* = 0.002; Figure 3). The 2-year OS rate was 97.6% for LI-negative patients compared to 90.7% for LI-positive patients, and the 5-year OS rate was 96.1% and 83.8%, respectively.

Multivariable Cox proportional hazards regression analysis, adjusting for age, sex, histological type, and tumour diameter, confirmed LI as an independent and highly significant adverse prognostic factor in this low-risk subgroup (HR: 4.002, 95% CI: 1.567–10.221, *p* = 0.004). The complete multivariable model results are presented in Table 5. The wide confidence interval reflects the limited number of events (*n* = 19) in this highly selected cohort.

## 4. Discussion

The diverse biological behaviour of NSCLC and the influence of numerous prognostic factors present challenges in standardising treatment approaches. Although LI is viewed as a critical step in tumour progression and the onset of metastasis [12], the inability of the standard TNM staging system to account for prognostic differences within the same stage is well-documented [4,5]. This retrospective cohort study, utilising a large series of 988 consecutive patients with robust long-term follow-up, aimed to definitively establish the independent prognostic role of LI, particularly focusing on its critical impact on early-stage patients currently ineligible for adjuvant therapy.

### 4.1. The General Prognostic Role of LI and Nodal Relationship

Our primary findings strongly support the adverse prognostic significance of LI. The presence of LI was significantly associated with markers of aggressive disease, including larger tumour size, advanced stage, and dramatically higher rates of regional nodal involvement (*p* < 0.001). This observation supports the hypothesis that LI represents the initial anatomical gateway for the metastatic cascade [13]. In multivariable analysis adjusting for age, sex, histology, tumour size, pathological stage, extent of resection, and calendar period, LI was confirmed as an independent predictor of worse OS (HR: 1.520, 95% CI: 1.004–2.301, *p* = 0.048).

### 4.2. The Critical and Independent Impact of LI in Early-Stage Patients

The most important and clinically relevant contribution of our study is the demonstration that LI has a profound and independent prognostic effect in the highly selected, low-risk subgroup of 347 early-stage patients (TNM Stage I, N0, no vascular/pleural invasion). These are precisely the patients for whom current international guidelines do not recommend adjuvant therapy [14].

In this homogeneous, low-risk cohort, the presence of LI led to a drastic survival divergence, with the 5-year OS rate declining from 96.1% (LI-negative) to 83.8% (LI-positive). Multivariable Cox regression analysis confirmed that LI is an independent adverse prognostic factor in this setting (HR: 4.002, 95% CI: 1.567–10.221, *p* = 0.004), after adjusting for age, sex, histological type, and tumour diameter. The wide confidence interval reflects the limited number of events (*n* = 19) in this highly selected population, and the finding should be interpreted with appropriate caution pending validation in larger prospective cohorts.

This finding is supported by other investigations; for example, Tamiya et al. similarly highlighted the critical importance of LI as a predictor of recurrence even in patients with Stage I disease [15]. These data suggest that the presence of LI acts as a “hidden risk factor” that current TNM staging fails to recognise [16], leading to prognostic divergence even in patients deemed low risk [17].

An alternative mechanistic explanation for the inferior survival observed in LI-positive N0 patients is the possibility of occult nodal micrometastases undetectable by standard H&E-based lymph node sampling. LI, as the anatomical first step in lymphatic dissemination, may reflect early nodal seeding below the threshold of conventional pathological detection, effectively representing a biologically node-positive phenotype in clinically N0 patients. This hypothesis underscores the potential value of more sensitive nodal evaluation techniques, such as molecular staging or serial sectioning with immunohistochemistry, in future prospective studies.

### 4.3. Relationship with Histological Subtype

Our findings clarify the biological basis of LI’s role, particularly in adenocarcinoma. LI was observed most frequently in the solid (53.2%; 66/124) and acinar (42.9%; 105/245) predominant patterns, compared to the indolent lepidic (19.4%) pattern (*p* < 0.001). This provides additional evidence that LI is an inherent expression of high-grade tumour biology and metastatic potential [18], aligning with reports such as Sugita et al., which found LI to be a poorer prognostic factor specifically in adenocarcinoma [19].

### 4.4. Clinical Implications and Future Directions

The presence of LI should be an integral part of the multidisciplinary treatment approach and considered in treatment planning, regardless of nodal status and disease stage. LI may identify a subset of early-stage N0 NSCLC patients who warrant closer postoperative surveillance and prospective evaluation for adjuvant treatment strategies, mirroring studies such as that by Tsutani et al., which suggested improved survival for high-risk LI-positive patients receiving chemotherapy [20]. However, the current study does not directly demonstrate a survival benefit from adjuvant therapy in LI-positive patients, and validation in prospective cohorts is required before LI can be formally integrated into staging algorithms or treatment guidelines. Furthermore, elucidating the molecular mechanisms of LI, such as the VEGF-C/D/VEGFR-3 axis [21], may contribute to identifying biological targets for this high-risk group.

### 4.5. Study Limitations

Our study’s retrospective design inherently carries certain limitations. First, consistent recurrence and cause-of-death data were not available across the full 17-year study period, precluding reliable calculation of disease-free survival, recurrence-free survival, or cancer-specific survival—endpoints that would more directly address the biological role of LI and the question of adjuvant therapy benefit. Second, the 17-year study period introduces potential treatment-era heterogeneity, including changes in staging practices, systemic therapy options, and surgical techniques. Although calendar period was included as a covariate in the multivariable model for the entire cohort—and was itself a highly significant prognostic factor (HR: 0.217, *p* < 0.001)—residual confounding from era-related changes cannot be fully excluded. Third, the limited number of events in the low-risk subgroup (*n* = 19) results in wide confidence intervals and warrants caution in interpreting the subgroup HR estimate. Fourth, lymphatic invasion was assessed using standard H&E staining without immunohistochemical confirmation (e.g., D2-40/podoplanin), and formal interobserver agreement statistics were not prospectively calculated, which may introduce some variability in LI classification. Measures such as including only patients who underwent R0 anatomical resection were taken to minimise confounding from variations in surgical technique.

## 5. Conclusions

This study demonstrates that the presence of lymphatic invasion (LI) is a significant and independent adverse prognostic factor for overall survival in resected NSCLC patients. The most critical finding is that LI acts as a powerful, hidden risk factor in the homogeneous subgroup of early-stage (TNM Stage I, N0) patients, in whom LI positivity leads to a substantial divergence in 5-year overall survival (96.1% vs. 83.8%) and independently increases the risk of mortality (multivariable HR = 4.002, 95% CI: 1.567–10.221) after adjustment for standard clinicopathological variables. Furthermore, analysis of adenocarcinoma subtypes revealed that LI exerted a more pronounced prognostic effect in the acinar and solid patterns. LI may identify a subset of early-stage N0 NSCLC patients who warrant closer postoperative surveillance and prospective evaluation for adjuvant treatment strategies. Validation in prospective cohorts is required before LI can be formally integrated into staging algorithms or treatment guidelines.

## Figures and Tables

**Table 1:** Clinicopathological Characteristics of the Entire Cohort Stratified by Lymphatic Invasion Status.

	LI (−) Mean ± SD/*n*	LI (+) Mean ± SD/*n*	*p*-Value
Tumour diameter (mm)	26.7 ± 21.5	33.9 ± 20.0	<0.001 *
Nodal Status			<0.001 †
N0	513	226	
N1	60	112	
N2	11	65	
N2a	10	56	
N2b	1	9	
N3	0	1	
Pathological Stage			<0.001 †
Stage 0	8	0	
Stage I	407	167	
Stage II	113	120	
Stage III	51	104	
Stage IV	5	13	

LI: lymphatic invasion; *: Student’s *t*-test; †: Chi-square test.

**Table 2:** Association of Lymphatic Invasion Status with Main Histological Subtypes of NSCLC.

Histological Subtype	LI (−)	LI (+)	*p*-Value
Adenocarcinoma	387 (61.9%)	238 (38.1%)	0.017 *
Squamous cell carcinoma	149 (56.9%)	113 (43.1%)	
Other	48 (47.5%)	53 (52.5%)	

LI: lymphatic invasion; NSCLC: non-small cell lung cancer; *: Chi-square test.

**Table 3:** Lymphatic Invasion Rates According to Histological Subtypes in the Adenocarcinoma Cohort.

Adenocarcinoma Subtype	LI (−)	LI (+)	*p*-Value
Solid	58 (46.8%)	66 (53.2%)	<0.001 *
Acinar	140 (57.1%)	105 (42.9%)	
Micropapillary	12 (50.0%)	12 (50.0%)	
Papillary	35 (76.1%)	11 (23.9%)	
Lepidic	50 (80.6%)	12 (19.4%)	
Mucinous	25 (75.8%)	8 (24.2%)	

LI: lymphatic invasion; *: Chi-square test. Analysis restricted to 534 cases successfully classified into one of the six predominant subtypes. The remaining 91 cases consisted of unclassifiable/rare variants and were excluded from this analysis.

**Table 4:** Multivariable Cox Proportional Hazards Regression Analysis—Entire Cohort (*n* = 965).

Variable	HR	95% CI	*p*-Value
Lymphatic invasion (positive vs. negative)	1.520	1.004–2.301	0.048
Age (per year)	1.024	1.004–1.044	0.016
Sex (female vs. male)	0.473	0.283–0.789	0.004
Histological type			0.173
Squamous vs. adenocarcinoma	1.464	0.933–2.297	0.098
Other vs. adenocarcinoma	1.509	0.870–2.618	0.143
Tumour diameter (per mm)	1.001	0.990–1.011	0.918
Pathological stage			0.005
Stage II vs. Stage I	1.022	0.596–1.752	0.937
Stage III–IV vs. Stage I	2.243	1.269–3.965	0.005
Extended resection (yes vs. no)	0.734	0.333–1.617	0.443
Calendar period (2015–2023 vs. 2006–2014)	0.217	0.145–0.326	<0.001

HR: hazard ratio; CI: confidence interval.

**Table 5:** Multivariable Cox Proportional Hazards Regression Analysis—Low-Risk Subgroup (*n* = 339).

Variable	HR	95% CI	*p*-Value
Lymphatic invasion (positive vs. negative)	4.002	1.567–10.221	0.004
Age (per year)	0.992	0.938–1.050	0.791
Sex (female vs. male)	0.686	0.236–1.997	0.490
Histological type			0.009
Squamous vs. adenocarcinoma	4.465	1.708–11.668	0.002
Other vs. adenocarcinoma	N/A *	—	0.986
Tumour diameter (per mm)	1.076	0.994–1.165	0.069

HR: hazard ratio; CI: confidence interval. * The “other histology” category contained 13 patients with no observed events; the HR estimate is unreliable and is not reported.

## Data Availability

The raw data supporting the conclusions of this article will be made available by the authors on request.
